# A Phaseless Source Reconstruction Method Based on Adam Optimization Algorithm Combined with Regularization

**DOI:** 10.3390/s26030939

**Published:** 2026-02-01

**Authors:** Zhangqiang Ma, Zhaowen Yan, Kunkun Hu, Fuyu Zhao, Jianhao Ge

**Affiliations:** School of Electronic and Information Engineering, Beihang University, Beijing 100191, China; by1902031@buaa.edu.cn (Z.M.); by2302128@buaa.edu.cn (F.Z.); by2402139@buaa.edu.cn (J.G.)

**Keywords:** EMC, equivalent dipole, Adam optimization, L2 regularization, source reconstruction

## Abstract

In the solution of equivalent dipoles for inverse electromagnetic problems, the traditional least squares method suffers from ill-conditioned matrices, resulting in insufficient accuracy and anti-noise performance, while existing optimization algorithms tend to fall into local optima during iteration. To address these issues, this paper proposes a phaseless source reconstruction method combining the Adam optimization algorithm with L2 regularization, which can stably solve the equivalent dipole source. The proposed method uses Adam optimization to avoid the direct inversion of ill-conditioned matrices, which improves the accuracy of near-field source reconstruction and effectively avoids falling into local optima. The introduced L2 regularization further suppresses local optima and significantly enhances the anti-noise performance of the equivalent dipole solution. In addition, simulations and experiments are carried out to verify the effectiveness of the proposed method.

## 1. Introduction

With the rapid development of electronic devices, electronic components and their assembled electronic systems are evolving toward miniaturization, high density, high speed, and high frequency, and these trends inevitably aggravate electromagnetic interference (EMI) issues, posing significant challenges to electromagnetic compatibility (EMC) design [1,2]. Accurate EMI evaluation and identification of interference sources are essential for effective mitigation. In recent years, near-field scanning technology has been widely adopted in engineering practice as an efficient, flexible, and programmable approach for EMI source identification [3,4]. The process of acquiring near-field electromagnetic information of the device under test (DUT) using electric and magnetic field probes, followed by establishing an equivalent radiation source model, is referred to as source reconstruction. Due to commercial confidentiality and module packaging constraints, obtaining detailed physical dimensions and field distributions of noise sources has become increasingly difficult, making research related to source reconstruction particularly practically significant [5].

Using near-field test data, the equivalent source model of the DUT can be extracted, which mainly includes two core forms: the equivalent current model and the equivalent dipole model [6,7]. The construction of the equivalent current model is theoretically based on integral functions and Green’s functions [8]. The equivalent dipole model is established relying on the plane wave spectrum theory and the source reconstruction principle, and realizes the equivalent substitution of the actual radiation source through a set of infinitesimal dipoles with different types, amplitudes, phases and spatial position characteristics [9,10,11]. This model has important application value in scenarios such as near-field to far-field transformation, quantitative evaluation of near-field EMI, and diagnosis of electromagnetic emission problems. In recent years, the dipole model has also evolved from a single form to collections of multiple dipoles, distributed arrays, and spherical harmonic multipole expansion forms with high-order components, adapting to scenarios such as multi-source detection and complex target modeling [12,13,14].

Traditional equivalent dipole source reconstruction methods require the simultaneous acquisition of amplitude and phase information of different near-field components [15]. Due to limitations in measuring tangential electric-field components, obtaining complete phase data is complex and prone to error, motivating phaseless source reconstruction methods [16,17]. In the absence of phase information, the linear system of equations to be solved during the source reconstruction process is transformed into a nonlinear system of equations. Regularization methods based on the least squares method have been employed to solve the equivalent dipole model, integrating Tikhonov regularization and truncated singular value decomposition (TSVD) regularization [16]. However, these traditional methods are relatively sensitive to ill-conditioned transformation matrices, and the greater the condition number of the matrix, the poorer the solution accuracy and anti-noise performance. Additionally, to address this challenge, various global optimization algorithms—including differential evolution, genetic algorithms, and particle swarm optimization (PSO) algorithm—have been explored. Refs. [18,19] applied the differential dynamic evolution algorithm to solve the equivalent dipole model, and Ref. [20] further proposed the differential evolution algorithm; Ref. [21] utilized the genetic algorithm for equation solving; the PSO algorithm and quantum particle swarm optimization (QPSO) algorithm have also been adopted in source reconstruction studies [22,23,24]. These optimization algorithms also tend to fall into local optima and thus struggle to solve electromagnetic source reconstruction problems in complex multi-modal scenarios.

To address the aforementioned issues, this paper proposes a phaseless source reconstruction method combining the Adam optimization algorithm and L2 regularization. By means of the Adam optimization algorithm, the method avoids the direct inversion of ill-posed matrices. It not only improves the accuracy of near-field source reconstruction but also effectively avoids local optimal solutions. Meanwhile, the introduction of L2 regularization constrains the optimization process, further suppresses local optimality, and enhances the anti-noise performance of equivalent dipole solution. The fusion of the two ultimately achieves stable and accurate phaseless source reconstruction of complex near-field electromagnetic fields. It has a wide range of applications, covering EMI source localization in consumer electronics and near-field reconstruction of 5G high-frequency devices in the field of electronics and communications, as well as multi-source magnetic target detection and near-field coupling prediction of complex equipment in the field of general electromagnetic detection. This method can effectively solve the EMC challenges in related fields and possesses significant practical application value.

The structure of this paper is organized as follows: Section 2 elaborates on the proposed method in detail; Section 3 designs two sets of numerical experiments to verify the feasibility and accuracy of the method; Section 4 conducts an analysis based on the measured data of physical samples to complete the verification of the method’s practical effectiveness; finally, Section 5 summarizes the full text and draws the research conclusions.

## 2. Proposed Method

### 2.1. Equivalent Dipole Source

**Figure 1:**
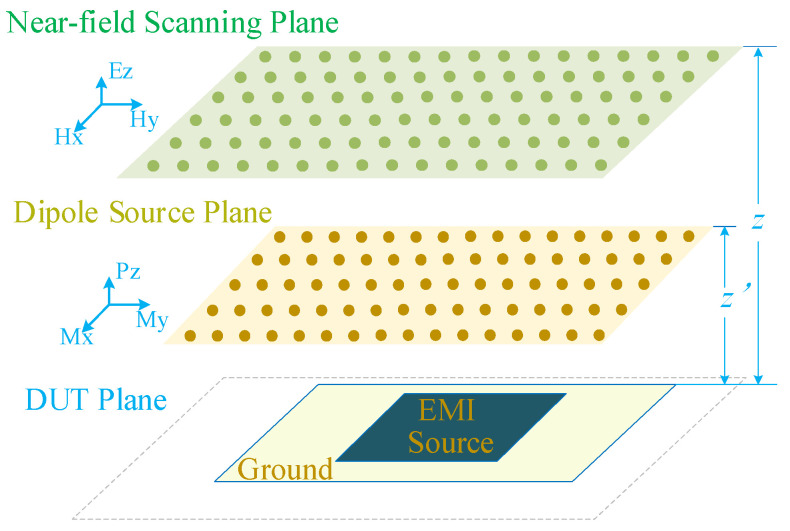
Equivalent dipole model based on near-field scanning.

In the Cartesian coordinate system, six fundamental equivalent dipoles are defined: electric dipoles (*P_x_*, *P_y_*, *P_z_*) and magnetic dipoles (*M_x_*, *M_y_*, *M_z_*) [25]. This work employs *M_x_*, *M_y_* and *P_z_* dipoles to represent the actual EMI source, as illustrated in Figure 1. The core rationale for selecting *M_x_*, *M_y_* and *P_z_* is based on three key considerations.

First, the electromagnetic radiation of a printed circuit board (PCB) mainly consists of two components. One is the magnetic field radiation generated by the loop currents formed by different traces on the PCB, which can be equivalent to *M_x_* and *M_y_*; the other is the electric field radiation induced by the potential difference between the PCB’s ground plane and metal structures such as traces, which can be equivalent to *P_z_*.

Second, developing high-performance tangential electric field probes that meet engineering requirements poses substantial challenges, resulting in high difficulty in measuring the tangential electric field in the near field [16]. Consequently, only the normal electric dipole *P_z_* is selected among electric dipoles. Compared with the two tangential electric dipoles, the adoption of a single normal electric dipole can reduce the computational complexity of source reconstruction while improving the extraction efficiency of the equivalent source.

Third, most PCBs contain only a single ground plane. Owing to the image theory, the near-field radiation intensity of the dipoles *M_x_*, *M_y_* and *P_z_* is significantly higher than that of the dipoles *P_x_*, *P_y_* and *M_z_* [19].

In this paper, the normal near electric field and tangential near magnetic field are selected to verify the effectiveness of the proposed method using equivalent dipoles. Given the known equivalent dipole sources [16], the electromagnetic field distribution at any arbitrary point in space can be calculated as:

(1) AX=F,

where the transformation matrix ***A*** serves as the mapping matrix between the electromagnetic field and the equivalent dipole sources, the matrix ***X*** denotes the equivalent dipole sources, and the matrix ***F*** represents the electromagnetic field distribution matrix at the observation points. The near-field distribution vector ***F*** includes the normal component of the electric field *E_z_* and the tangential components of the magnetic field *H_x_* and *H_y_*. The equivalent dipole vector ***X*** consists of the electric dipole *P_z_* and the magnetic dipoles *M_x_* and *M_y_*. The expanded form of (1) is given in Appendix A.

### 2.2. Adam Optimization Algorithm

A method for solving the electromagnetic inverse problem based on the Adam optimization algorithm is proposed to invert the source distribution from the measured electromagnetic field data. Its core logic resides in minimizing the mean square error (MSE) between the calculated field and the measured field through iterative optimization, thereby solving for the equivalent dipole ***X*** in (1). The specific implementation process comprises three steps.

The first step is initialization of data and variables. The equivalent dipole ***X*** is initialized as a zero vector, and the parameters of the Adam optimizer are configured. Meanwhile, the maximum number of iterations and convergence threshold are defined. The optimization parameters include the learning rate (*α*), first-order momentum coefficient (*β*_1_), second-order momentum coefficient (*β*_2_), and numerical stability term (*δ*). The learning rate, a core parameter controlling the step size of parameter updates, is defined as the scaling factor for parameter adjustments in each iteration. For nonlinear electromagnetic field inverse problems, a smaller learning rate can be selected to mitigate the impact of numerical instability. The first-order momentum coefficient is used to accumulate the exponential moving average (EMA) of gradients, reflecting the inertia of the parameter update direction. The second-order momentum coefficient accumulates the EMA of squared gradients, characterizing the scale properties of gradients. The numerical stability term is an extremely small positive value that ensures numerical stability while exerting a negligible influence on gradient scales, thus safeguarding the numerical accuracy of the optimization process. For nonlinear problems such as electromagnetic field inversion, appropriate parameter values enable efficient convergence to a reliable solution while ensuring numerical stability.

The second step is iterative optimization process. In each iteration, the calculated field is generated by the matrix multiplication of the transformation matrix ***A*** and the current equivalent dipole ***X***. Subsequently, the residual ***r*** between the calculated field values and the measured field is calculated as follows:

(2) $$r=F_{\mathrm{cal}}-F=AX-F,$$

where ***F***_cal_ denotes the calculated field values, ***r*** represents the residual between the calculated field values and the measured field. It should be noted that the field values ***F***_cal_ and ***F*** are only amplitude values without phase information.

Meanwhile, the MSE loss function *L_MSE_* is calculated as follows:

(3) $$L_{MSE}=\frac{1}{2}{\left\|r\right\|}^{2},$$

where *L_MSE_* is the MSE loss function. $\left\|\cdot \right\|$ denotes the Euclidean norm of a matrix.

Further, the gradient of the loss function with respect to the equivalent source is derived by the transpose operation of the residual and the transformation matrix, which is computed as (4).

(4) $$g=A^{⊤}r,$$

where ***g*** stands for the gradient of the loss function with respect to the equivalent source.

Finally, the equivalent dipole source ***X*** is iteratively updated using the momentum update rules of the Adam algorithm, as given by follows:

(5) $$m_{t}=\beta_{1}m_{t-1}+(1-\beta_{1})g,$$

(6) $$v_{t}=\beta_{2}v_{t-1}+(1-\beta_{2})g⊙g,$$

(7) $${\hat{m}}_{t}=m_{t}/(1-\beta_{1}^{t}),$$

(8) $${\hat{v}}_{t}=v_{t}/(1-\beta_{2}^{t}),$$

(9) $$X_{t}=X_{t-1}-\alpha \cdot {\hat{m}}_{t}/(\sqrt{{\hat{v}}_{t}}+\delta ),$$

where ***m****_t_* is the first-order momentum estimate at the t-th iteration, which serves to smooth the gradient update direction. ***v****_t_* is the second-order momentum estimate at the t-th iteration, utilized for adaptively adjusting the learning rate. ${\hat{m}}_{t}$ and ${\hat{v}}_{t}$ are the bias correction terms for the first-order and second-order moments, respectively, designed to alleviate the bias issue of momentum estimates in the early stages of iteration. *t* denotes the number of iterations. ⊙ denotes the Hadamard product between matrices, also known as element-wise multiplication.

The third step is convergence criterion. The iteration is terminated when the difference in the loss function between adjacent iterations is less than the convergence threshold. Finally, the equivalent dipole ***X*** is obtained through inversion.

The Adam optimization algorithm stabilizes gradient fluctuations through momentum terms and avoids direct inversion of transformation matrix A. This effectively reduces the interference of ill-posed matrices with the solution results, improves the accuracy of the reconstruction field, and also effectively avoids local optimal solutions.

### 2.3. L2 Regularization

In electromagnetic field inversion problems, the condition number of the transformation matrix ***A*** is generally large, resulting in multiple sets of equivalent dipoles ***X*** that approximately satisfy the measured data in the solution space. Some of these solutions may contain physically unreasonable sharp fluctuations, leading to local optimization of the solution. L2 regularization imposes a penalty on the equivalent source ***X*** by introducing a regularization parameter *λ*: the value of *λ* directly determines the strength of the penalty term. By constraining the overall energy of the equivalent source ***X***, L2 regularization confines the solution to a physically reasonable compact subspace, effectively suppressing numerical instability caused by ill-posedness. When *λ* is too small, the constraint is ineffective, causing overfitting; when *λ* is too large, excessive smoothing leads to underfitting. The solution becomes overly smoothed, losing details of the true source.

The integration of L2 regularization into the Adam optimization algorithm primarily involves three key stages: modifying the objective function, adjusting the gradient calculation, and influencing parameter updates.

First, the loss function changes from *L_MSE_* to *L_λ_*, and the calculation formula is updated from (3) to (10). The newly added term $\frac{\lambda }{2}{\left\|X\right\|}^{2}$ acts as the regularization functional, where the regularization coefficient *λ* regulates its weight in the overall objective by scaling the value of the functional, thus guiding the optimization direction.

(10) $$L_{\lambda }=\frac{1}{2}{\left\|r\right\|}^{2}+\frac{\lambda }{2}{\left\|X\right\|}^{2},$$

Second, the parameter update of the Adam algorithm relies on the gradient field of the objective function. After differentiating the regularized objective function, the total gradient function changes from ***g*** to $g_{\lambda }$, and the calculation formula is updated from (4) to (11). The newly added term *λ**X*** is the regularization gradient, which imposes a constraint-guided effect on the solution.

(11) $$g_{\lambda }=A^{⊤}r+\lambda X,$$

Finally, the Adam algorithm achieves parameter update through the bias correction of the first-order momentum and second-order moment. The forms of its iterative formulas (5)–(9) remain unchanged; it only requires replacing the unregularized gradient ***g*** with the regularized gradient $g_{\lambda }$. The integration of regularization results in the update step size being subjected to stronger reverse correction, ultimately leading to the dynamic regularization of the solution.

**Figure 2:**
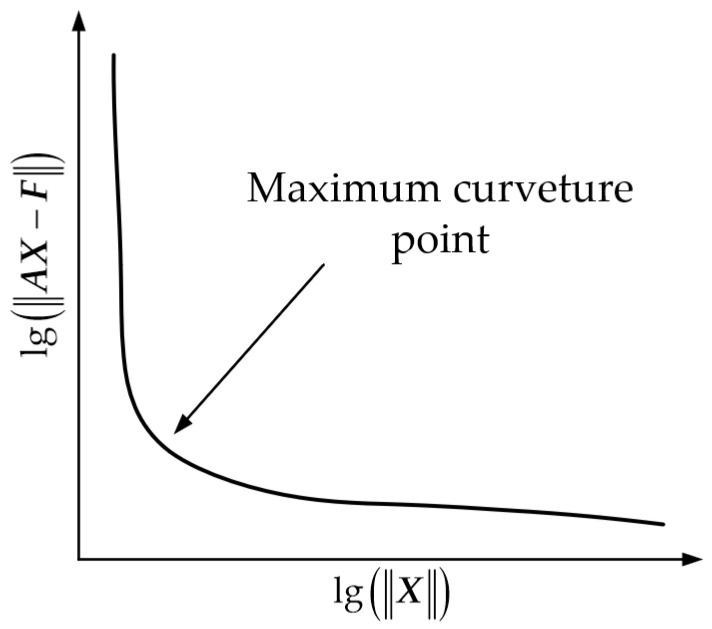
L-curve for obtaining an appropriate *λ*.

In each iteration, we adopt the L-curve method to determine an appropriate value of *λ* [16]. We iterate over *λ* values within a predefined range and compute the corresponding $\left\|X\right\|$ and $\left\|AX-F\right\|$. Then, as illustrated in Figure 2, we plot the L-curve on a logarithmic scale, with $\mathrm{lg}\left(\left\|X\right\|\right)$ as the abscissa and $\mathrm{lg}\left(\left\|AX-F\right\|\right)$ as the ordinate. The *λ* corresponding to the maximum curvature point is selected as the regularization parameter for the current iteration, thus achieving the dynamic adaptation of *λ* throughout the iteration process.

**Figure 3:**
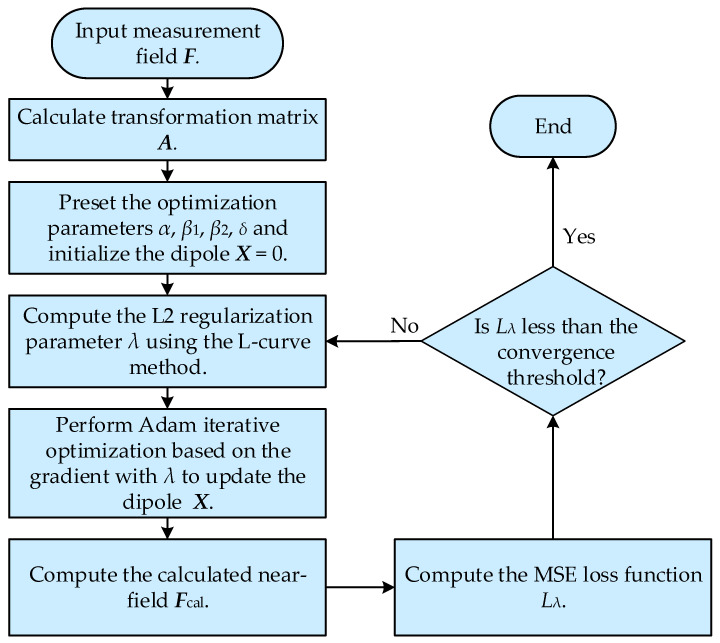
Flowchart of the proposed method.

Mathematically, the penalty mechanism formed by introducing the L2 regularization parameter *λ* not only enhances the anti-noise performance of electromagnetic field inversion but also effectively prevents the optimization process from falling into local optima. By integrating L2 regularization with the Adam optimizer, the proposed method not only achieves high accuracy and strong anti-noise performance but also effectively avoids convergence to local minima. The flow chart of the proposed method is shown in Figure 3.

The computational complexity of the proposed method is mainly determined by two key factors: the number of iterations and the dimension of optimization variables. The algorithm achieves the required accuracy after a fixed number of iterations, which is a constant value in our method. The dimension of optimization variables is only related to the number of equivalent dipoles and their amplitudes, denoted as N. The core computation in each iteration involves matrix operations and gradient updates based on the Adam optimization algorithm, resulting in a time complexity of O(K × N^2^), where K represents the number of iterations. In terms of space complexity, it is dominated by the storage of dipole amplitudes, gradient information, and intermediate calculation results, with a space complexity of O(N). Regarding scalability, the proposed method exhibits excellent adaptability: when the number of dipoles (N) increases or the number of measurement points expands to adapt to more complex near-field scenarios, the time complexity grows moderately with N^2^, which is fully manageable with current computing resources. Meanwhile, the fixed number of iterations avoids excessive complexity growth caused by additional iterations, ensuring that the method can be scaled to practical large-scale near-field source reconstruction tasks.

## 3. Simulation Examples and Validation

In this section, the effectiveness of the proposed method is verified through multiple repeated simulations and calculations by combining two simulation-based numerical examples. The proposed method is implemented on a computer equipped with an Intel Xeon Gold 6146 3.20 GHz CPU and 128 GB RAM.

### 3.1. Application in Patch Antenna

**Figure 4:**
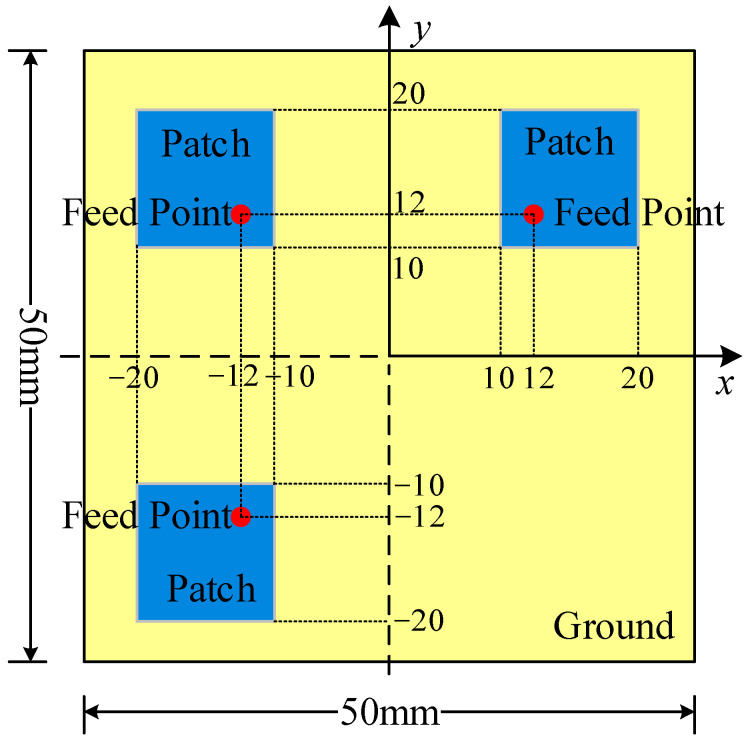
The patch antenna model.

The first simulation example consists of three 10 mm × 10 mm patch antennas, with the model illustrated in Figure 4. The dielectric substrate is duroid 5880 with a thickness of 3.2 mm, and the ground plane measures 50 mm × 50 mm. The positions of the three feeding points are (12 mm, 12 mm), (−12 mm, 12 mm) and (−12 mm, −12 mm), respectively, and the operating frequency is set to 4 GHz. The near-field observation plane is a 50 mm × 50 mm area located 4 mm directly above the patch antennas, while the dipole plane is deployed in the same area at a height of 1.5 mm. The number of observation points is 26 × 26 with a spacing of 4 mm between adjacent points, and the number of dipoles is 21 × 21 with an interval of 5 mm between each dipole.

#### 3.1.1. Parameter Analysis

The maximum number of iterations is set to 2000, and the convergence threshold is configured as 1 × 10^−8^. The optimization hyperparameters are preset as follows: the learning rate *α* is 1 × 10^−5^, the first-order momentum coefficient *β*_1_ is 0.90, the second-order momentum coefficient *β*_2_ is 0.999, and the numerical stability term *δ* is 1 × 10^−8^. The optimal values of the aforementioned hyperparameters are determined by comparing the iterative convergence curves generated under different parameter configurations.

**Figure 5:**
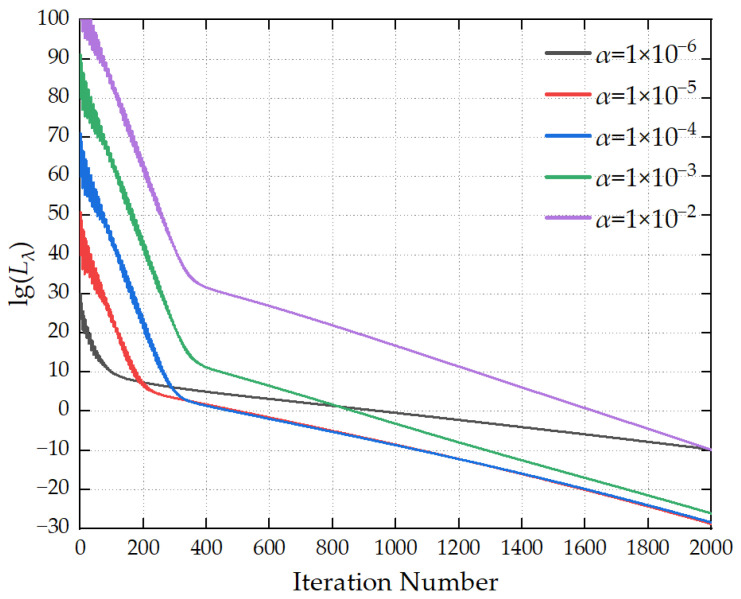
Convergence curves for different α values.

Figure 5 illustrates the variation in the loss function with the number of iterations under different *α* values. When *α* is excessively large, the initial loss function value remains high, resulting in a still elevated loss function value at the end of iterations. When *α* is too small, the loss function converges slowly, leading to low iteration efficiency. When *α* is set to a moderate value, a balance is achieved between the initial loss function value and the convergence speed. It is recommended that the value range of *α* be set to 1 × 10^−5^∼1 × 10^−4^.

**Figure 6:**
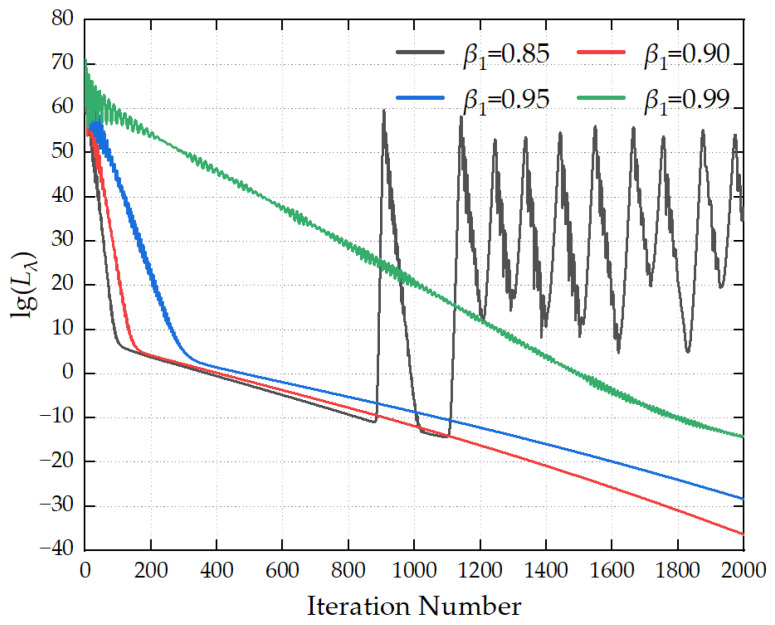
Convergence curves for different *β*_1_ values.

Figure 6 illustrates the influence of different first-order momentum decay coefficients *β*_1_ on the convergence process of the loss function. When *β*_1_ is excessively large, the loss curve tends to exhibit severe oscillations, which results in the non-convergence of the loss function. When *β*_1_ is too small, the loss function decreases at an overly slow rate, leading to low solution accuracy. When *β*_1_ is assigned a moderate value, a favorable balance is achieved between convergence speed and convergence stability. It is recommended that the value of *β*_1_ be set to 0.9.

**Figure 7:**
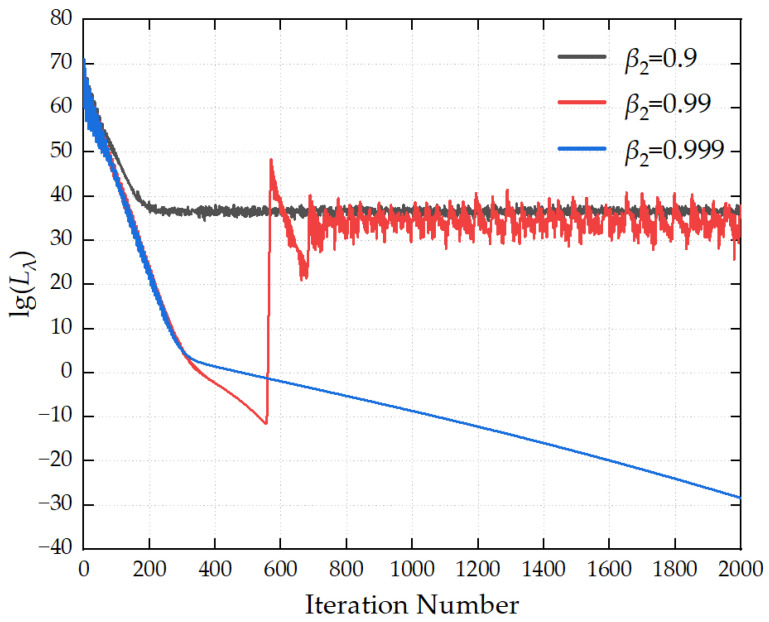
Convergence curves for different *β*_2_ values.

Figure 7 illustrates the influence of different second-order momentum decay coefficients *β*_2_ on the convergence process of the loss function. When *β*_2_ takes a relatively large value, the loss curve declines rapidly in the early stage of iterations and can achieve continuous convergence. When *β*_2_ is too small, the loss function tends to fall into oscillations and fails to converge. It is recommended that the value of *β*_2_ be set to 0.999.

**Figure 8:**
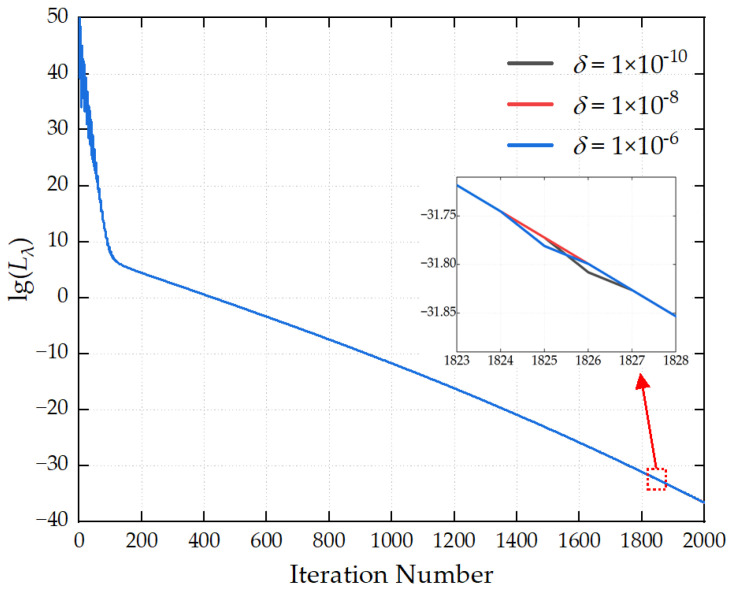
Convergence curves for different *δ* values.

Figure 8 demonstrates the influence of different numerical stability parameters *δ* on the convergence process of the loss function. The three curves in the figure almost completely overlap, and there are no significant differences in the initial values, decline rates, and final convergence accuracy of the loss function. As long as *δ* lies within a reasonable small value range, it will not exert a noticeable impact on the convergence performance. It is recommended that the value of *δ* be set to 1 × 10^−8^.

The amplitude values of the simulated field for the patch antenna model range from 1 × 10^−2^ to 40. From the convergence curves obtained under the influence of different optimization parameters mentioned above, it can be concluded that the loss function of the proposed method has already become very small when the number of iterations reaches 2000. At this point, the loss function is close to 1 × 10^−4^, which is 2 to 3 orders of magnitude smaller than the simulated field amplitude values, indicating that the source reconstruction accuracy is quite high within the limited number of iterations. The convergence threshold is defined as the difference between the loss functions of two adjacent iterations, which is generally 2 to 4 orders of magnitude smaller than the loss function itself. Therefore, the number of iterations is set to 2000 and the convergence threshold is set to 1 × 10^−8^.

In summary, to achieve high-precision source reconstruction, it is recommended that the number of iterations be set to 2000, the convergence threshold to 1 × 10^−8^, the learning rate to 1 × 10^−5^, the first-order momentum coefficient *β*_1_ = 0.9, the second-order momentum coefficient *β*_2_ = 0.999, and the numerical stability term *δ* = 1 × 10^−8^.

#### 3.1.2. Global Optimization

The proposed method features the core characteristic of global optimization. When addressing multimodal optimization problems, local optimization algorithms are often constrained by local information in the solution space and prone to trapping in local optimal solutions, making it difficult to achieve globally optimal solutions. The antenna model adopted in this study has a radiated field with distinct multimodal distribution characteristics, which can serve as a typical test case for verifying algorithm performance and effectively demonstrate the feasibility and superiority of the proposed method.

**Figure 9:**
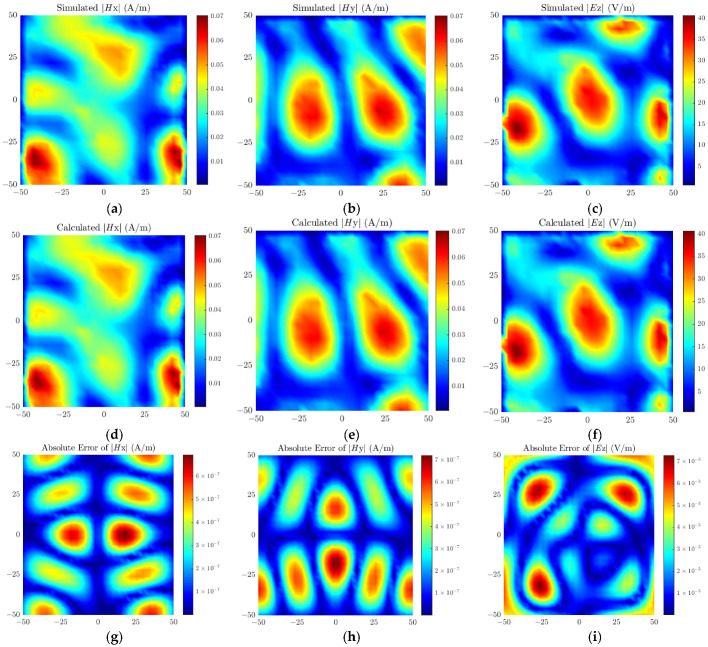
Simulated field values of (**a**) ***H****_x_*, (**b**) ***H****_y_*, (**c**) ***E****_z_*; Calculated field values of (**d**) ***H****_x_*, (**e**) ***H****_y_*, (**f**) ***E****_z_*; Absolute errors of (**g**) ***H****_x_*, (**h**) ***H****_y_*, (**i**) ***E****_z_* for the patch antenna.

Figure 9 presents the simulated field, calculated reconstructed field, and the distribution of absolute errors between the two for the antenna model, where the absolute error is defined as the amplitude difference between the simulated field and the calculated field. As can be seen from Figure 9, the amplitude of the absolute error is five orders of magnitude lower than that of the simulated field, which fully indicates that the source reconstruction accuracy of the proposed method reaches a high level. Meanwhile, the errors corresponding to each peak region in the field distribution are maintained within an extremely small range, further verifying the excellent global optimization performance of the proposed method, which holds broad prospects for engineering applications.

#### 3.1.3. Accuracy

To address the issue of overall error distortion caused by the amplification of relative error in weak signals, a weighted relative error (*ε*_wr_) is defined as (12) to quantitatively analyze the effectiveness of the proposed method. This relative error weights the error at each measurement point using the field value of that point, achieving weighted enhancement of strong signals and weighted reduction in weak signals. It can objectively reflect the reconstruction accuracy of the effective source region and is more referenceable in practical measurements.

(12) $$\varepsilon_{\mathrm{wr}}=\frac{\sum_{i,j}\left|F_{\mathrm{sim}}(i,j)\right|\frac{\left|F_{\mathrm{sim}}(i,j)-F_{\mathrm{cal}}(i,j)\right|}{\left|F_{\mathrm{sim}}(i,j)\right|+\epsilon }}{\sum_{i,j}\left|F_{\mathrm{sim}}(i,j)\right|}=\frac{\sum_{i,j}\left|F_{\mathrm{sim}}(i,j)-F_{\mathrm{cal}}(i,j)\right|}{\sum_{i,j}\left|F_{\mathrm{sim}}(i,j)\right|},$$

where ***F***_sim_ denotes the measured field from the true source or simulated source, and ***F***_cal_ represents the reconstructed field from the equivalent dipole source, both of which consist of three electromagnetic field components. (*i*, *j*) is the two-dimensional grid row and column index of the measurement point. *ε* is a tiny constant that provides robust correction for weak signals and noise points. The range of *ε*_wr_ is (0, 100%); the closer its value is to 0, the better the performance of the reconstructed field.

To demonstrate the accuracy of the proposed method, a comparison is conducted with the Adam optimization algorithm with regularization and a traditional least squares method. The traditional method selected is the phaseless source reconstruction method combining Tikhonov regularization and TSVD regularization proposed in [16]. By establishing dipoles at different plane heights to generate reconstructed fields, comparisons of weighted relative error *ε*_wr_ under various conditions are obtained, as presented in Table 1. The weighted relative error of the proposed method is significantly smaller than that of the traditional method. Meanwhile, it can be observed that when the height of the equivalent dipoles is set within the range of 1.5 mm to 3 mm, the source reconstruction error is minimal.

#### 3.1.4. Anti-Noise Performance

The correlation coefficient *ρ* is defined as (13), which serves to quantitatively evaluate the similarity between the calculated field and the simulated field. The value range of *ρ* is (−1, 1); the closer this value is to 1, the better the source reconstruction performance.

(13) $$\rho =\frac{\mathrm{Cov}\left(\left|F_{\mathrm{sim}}\right|,\left|F_{\mathrm{cal}}\right|\right)}{\sqrt{\mathrm{Var}\left(\left|F_{\mathrm{sim}}\right|\right)\mathrm{Var}\left(\left|F_{\mathrm{cal}}\right|\right)}},$$

where Cov denotes the covariance operation of matrices, and Var denotes the variance operation of matrices.

Near-field measurements are susceptible to external noise interference, where the measured data are a mixture of the true field values and noise. Therefore, the research on source reconstruction methods must take anti-noise performance into account. The simulated field is regarded as a pure noise-free reference, and the anti-noise performance of source reconstruction methods is compared between this reference and the simulated field injected with white Gaussian noise of varying intensities. White Gaussian noise is commonly encountered in engineering; the noise intensity is set to be correlated with the amplitude of the simulated field, calculated as follows:

(14) $$N_{F}=L_{n}\cdot N\left(0,1\right)⊙F_{\mathrm{pure}},$$

where ***F***_pure_ is the pure magnetic field matrix, ***N****_F_* is the generated white Gaussian noise matrix, and $N\left(0,1\right)$ is a random matrix following a normal distribution. The three matrices are of the same dimension. *L_n_* is the noise level coefficient, which is used to adjust the relative magnitude of the noise. Noise level coefficients of 2%, 5%, 10%, 20%, 30%, and 40% are selected to compare the anti-noise performance of different methods.

White Gaussian noise with different intensities is injected into the pure simulated field; source reconstruction is performed separately to obtain the calculated field. All similarity indices are calculated as the similarity between the calculated field and the pure simulated field under different noise levels. By comparing the attenuation amplitude of similarity under different noise levels, the anti-noise performance of the proposed source reconstruction method is quantitatively evaluated.

The comparison results of source reconstruction similarity under different noise levels are presented in Table 2. As can be seen from Table 2, at noise levels below 10%, the anti-noise performance of the proposed method and the basic Adam optimization method is significantly superior to that of the traditional method, with the similarity decreasing by less than 2%. Meanwhile, as the noise level increases, the anti-noise performance of the proposed method becomes increasingly superior to that of the basic Adam optimization method. By introducing a regularization coefficient into Adam optimization, the sensitivity of source reconstruction to noise interference is effectively suppressed. L2 regularization can improve the anti-noise performance of source reconstruction methods and thus can be effectively applied to phase-free source reconstruction in electromagnetic near-field measurements.

### 3.2. Simulation of Power Divider

**Figure 10:**
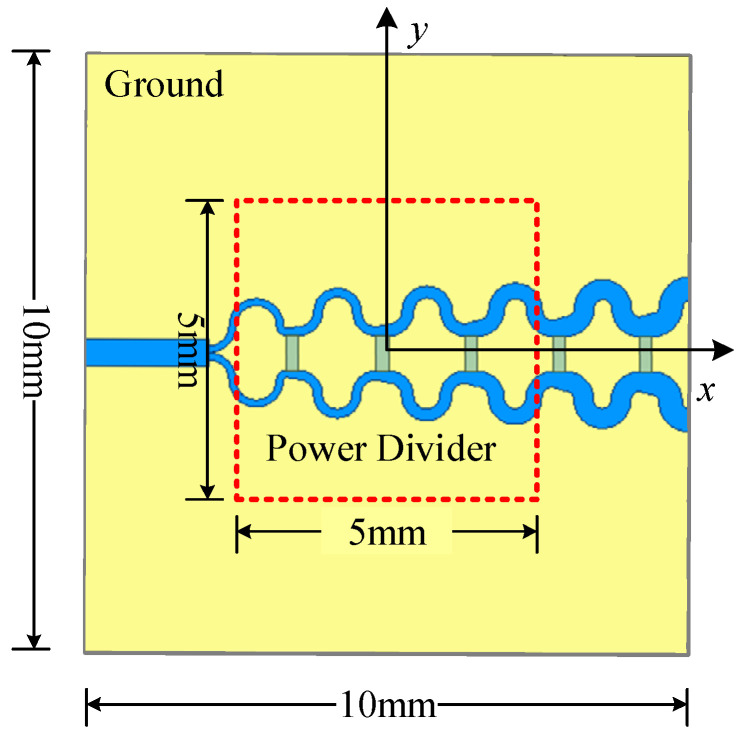
The power divider model.

**Figure 11:**
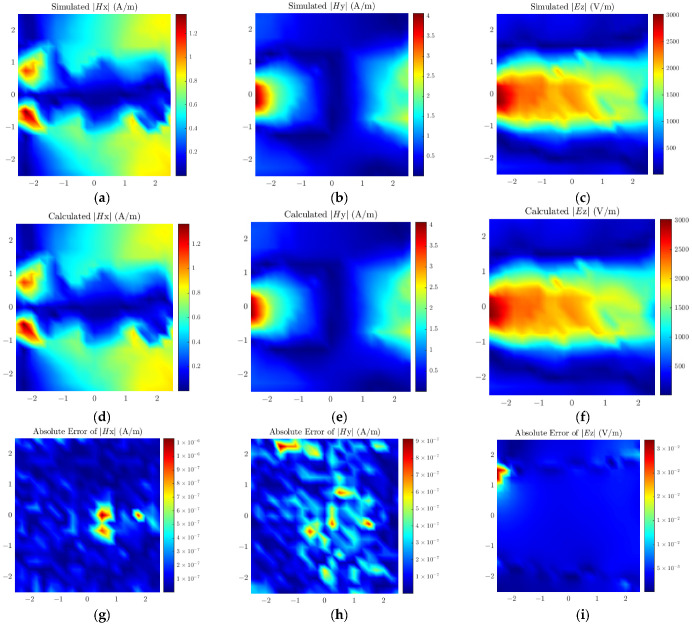
Simulated field values of (**a**) ***H****_x_*, (**b**) ***H****_y_*, (**c**) ***E****_z_*; Calculated field values of (**d**) ***H****_x_*, (**e**) ***H****_y_*, (**f**) ***E****_z_*; Absolute errors of (**g**) ***H****_x_*, (**h**) ***H****_y_*, (**i**) ***E****_z_* for the power divider.

The proposed method is verified through a simulated power divider, as illustrated in Figure 10. The PCB dimensions are 10 mm × 10 mm, and the dielectric substrate is FR4 with a thickness of 3 mm. The three terminal impedances of the power divider are all set to 50 Ω, and the input power of the unbalanced port is set to 1 VA. The selected region is a 5 mm × 5 mm area 1 mm above the transmission line, where the near-field data is collected. The number of observation points is 21 × 21, with a spacing of 0.25 mm between adjacent observation points. The dipole height is set to 0.5 mm; the number of dipoles is 19 × 19, and the spacing between adjacent dipoles is 0.26 mm. Figure 11 presents the simulated field, calculated field, and the distribution of absolute errors between the two for the power divider model. Similarly, Table 3 lists the weighted relative error for both methods, showing that the proposed method outperforms the traditional method in accuracy.

## 4. Measurement Examples and Validation

**Figure 12:**
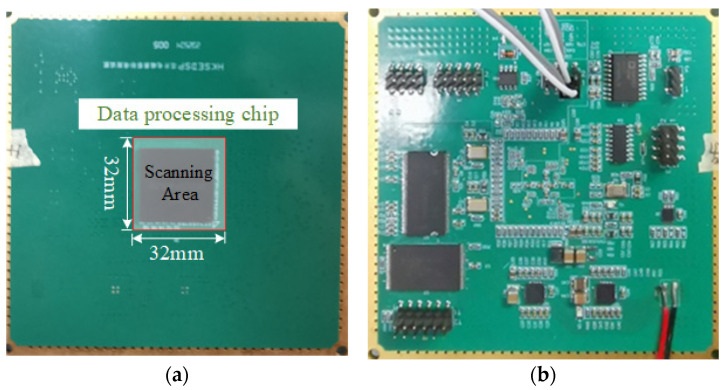
Front (**a**) and back (**b**) sides of the data processing chip test board.

In this section, physical verification of the proposed method is conducted. A test board of a data processing chip is selected as the DUT, and the front and back sides of the main board are illustrated in Figure 12. The DUT is measured to verify the effectiveness and accuracy of the proposed method.

The test procedure for electromagnetic near-field radiation emission is as follows: The DUT is powered by a DC power supply and excited by a signal generator. The near-field probe is vertically placed 1 mm above the DUT and moved point-by-point in the two-dimensional plane above the DUT via a robotic arm. The near-field probe converts the field signal into an electrical signal, which is read and recorded by a spectrum analyzer connected through a coaxial cable. Both the electric field probe and magnetic field probe used in the measurement were pre-calibrated [26,27].

**Figure 13:**
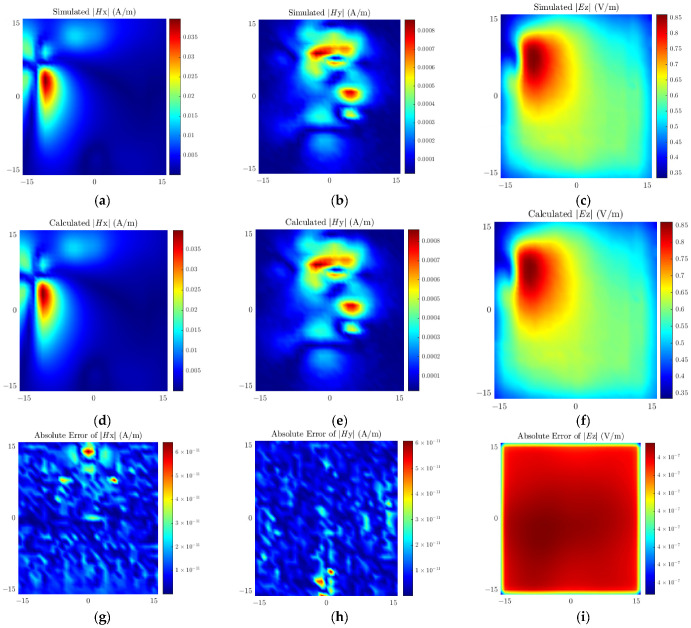
Measured field values of (**a**) ***H****_x_*, (**b**) ***H****_y_*, (**c**) ***E****_z_*; Calculated field values of (**d**) ***H****_x_*, (**e**) ***H****_y_*, (**f**) ***E****_z_*; Absolute errors of (**g**) ***H****_x_*, (**h**) ***H****_y_*, (**i**) ***E****_z_*.

The near-field scanning area is a 32 mm × 32 mm region 1 mm directly above the chip. The number of scanning points is 33 × 33, with a spacing of 1 mm between adjacent scanning points. The dipole source plane is set at a height of 0.5 mm; the number of dipoles is set to 25 × 25, and the spacing between adjacent dipoles is 1.33 mm. The scanned near-field and the near-field generated by the equivalent dipole sources are shown in Figure 13. The comparison between the proposed method and the traditional method is presented in Table 4, demonstrating that the dipole model of the proposed method has higher accuracy than that of the traditional method.

## 5. Conclusions

This study proposes a phaseless source reconstruction method integrating the Adam optimization algorithm with L2 regularization, which effectively overcomes the limitations of traditional methods and other optimization algorithms in solving near-field inverse problems. Verified quantitatively through numerical simulations and physical measurements, the method exhibits excellent performance in reconstruction accuracy, anti-noise capability, and global optimization performance. In terms of reconstruction accuracy, the weighted relative error *ε*_wr_ of the proposed method can be as low as 0.0001%, and the absolute error is five orders of magnitude smaller than the amplitude of the measured field. Regarding anti-noise performance, when the noise level coefficient does not exceed 10%, the decrease in the correlation coefficient *ρ* is less than 2%, demonstrating extremely strong anti-noise performance. Additionally, the method has broader applicability compared with other optimization methods—even in complex scenarios with multimodal near-field distributions, the reconstruction error in each peak region remains minimal, enabling accurate field reconstruction.

However, the parameter selection of the Adam optimization algorithm is crucial; improper configuration may lead to failure to converge during iterations. Future research can integrate big data and neural networks to realize automatic optimal parameter selection without manual debugging, further enhancing the engineering practicality and efficiency of the method.

The dipole source reconstruction method proposed in this study, with its excellent anti-noise performance and superior reconstruction accuracy, is suitable for moderate-complexity near-field tasks such as EMI source localization in consumer electronics and the near-field reconstruction of 5G devices. It should be noted, however, that the proposed method does not incorporate higher-order momentum sources, which limits its broader applicability. Spherical harmonic expansion can decompose complex electromagnetic sources into multipole components including dipoles and quadrupoles. In the future, our method can draw on multi-source modeling strategies to improve the existing Adam optimization algorithm framework, enabling it to simultaneously optimize the amplitudes of both dipole and quadrupole components, thereby developing a near-field source reconstruction method applicable to more complex scenarios such as aerospace, automotive, and marine engineering fields.

## Figures and Tables

**Table 1:** Weighted relative error under different dipole heights.

Dipole Height (mm)	*ε*_wr_ of The Traditional Method	*ε*_wr_ of The Proposed Method
0.5	4.94%	1.03%
1.0	3.86%	0.77%
1.5	2.31%	0.0001%
2.0	3.11%	0.0012%
2.5	2.42%	0.0002%
3.0	2.67%	0.0003%

**Table 2:** Comparison of source reconstruction similarity under different noise levels.

Noise Level Coefficient *L_n_*	*ρ* of The Traditional Method	*ρ* of Adam Optimization	*ρ* of The Proposed Method
0%	98.92%	100%	100%
2%	96.35%	99.92%	99.94%
5%	93.78%	99.20%	99.61%
10%	92.49%	98.07%	98.44%
20%	85.43%	92.29%	94.21%
30%	77.21%	87.58%	89.21%
40%	74.83%	78.92%	81.76%

**Table 3:** Weighted relative error of the two methods for the power divider.

Method	*ε*_wr_
the traditional method	6.94%
the proposed method	0.0003%

**Table 4:** Weighted relative error of the two methods for the measurement example.

Method	*ε*_wr_
the traditional method	2.31%
the proposed method	0.0001%

## Data Availability

Data are contained within the article.

## Abbreviations

The following abbreviations are used in this manuscript:

EMI	Electromagnetic interference
EMC	Electromagnetic compatibility
DUT	Device under test
TSVD	Truncated singular value decomposition
PSO	Particle swarm optimization
QPSO	Quantum particle swarm optimization
PCB	printed circuit board
MSE	Mean square error
EMA	Exponential moving average

## Appendix A

The expanded form of (1) is given by:

(A1) $$\left(\begin{array}{ccc} A_{PzEz} & A_{MxEz} & A_{MyEz}\\ A_{PzHx} & A_{MxHx} & A_{MyHx}\\ A_{PzHy} & A_{MxHy} & A_{MyHx} \end{array}\right)\left(\begin{array}{c} P_{z}\\ M_{x}\\ M_{y} \end{array}\right)=\left(\begin{array}{c} E_{z}\\ H_{x}\\ H_{y} \end{array}\right).$$

The submatrices of the mapping matrix ***A*** represent the contributions of different dipoles to various field distributions. For instance, *A_PzEz_* denotes the relationship between the electric dipole *P_z_* and the tangential electric field component *E_z_* in space. The field distribution at each observation point can be calculated as the superposition of the corresponding fields generated by all dipoles and their images. Each sub-mapping matrix is given by follows:

(A2) $$A_{PzEz}=\frac{\eta }{2\pi }\left[\frac{f_{1}(r){\left(z-z^{\prime }\right)}^{2}}{r^{3}}+\frac{f_{1}(r^{\prime }){\left(z+z^{\prime }\right)}^{2}}{{\left(r^{\prime }\right)}^{3}}\right.\left.-\frac{jk({\left(x-x^{\prime }\right)}^{2}+{\left(y-y^{\prime }\right)}^{2})}{2}\left(\frac{f_{2}(r)}{r^{2}}+\frac{f_{2}(r^{\prime })}{{\left(r^{\prime }\right)}^{2}}\right)\right],$$

(A3) $$A_{MxEz}=\frac{-jk}{4\pi }\left[\frac{f_{1}(r)(y-y^{\prime })}{r}+\frac{f_{1}(r^{\prime })(y-y^{\prime })}{r^{\prime }}\right],$$

(A4) $$A_{MyEz}=\frac{jk}{4\pi }\left[\frac{f_{1}(r)(x-x^{\prime })}{r}+\frac{f_{1}(r^{\prime })(x-x^{\prime })}{r^{\prime }}\right],$$

(A5) $$A_{PzHx}=-\frac{jk}{4\pi }\left(\frac{f_{1}(r)}{r^{2}}+\frac{f_{1}(r^{\prime })}{{\left(r^{\prime }\right)}^{2}}\right)\cdot (y-y^{\prime }),$$

(A6) $$\begin{array}{c} A_{MxHx}=\frac{1}{2\pi \eta }\frac{\left(x-x^{\prime })(y-y^{\prime }\right)}{{\left[{\left(x-x^{\prime }\right)}^{2}+{\left(y-y^{\prime }\right)}^{2}\right]}^{1/2}}\left[\frac{\left(z-z^{\prime })f_{1}(r\right)}{r^{4}}\right.\left.+\frac{\left(z+z^{\prime })f_{1}(r^{\prime }\right)}{{\left(r^{\prime }\right)}^{4}}\right]+\frac{jk}{4\pi \eta }\frac{y-y^{\prime }}{{\left[{\left(x-x^{\prime }\right)}^{2}+{\left(y-y^{\prime }\right)}^{2}\right]}^{1/2}}\\ \left[\frac{{\left[{\left(x-x^{\prime }\right)}^{2}+{\left(z-z^{\prime }\right)}^{2}\right]}^{1/2}(z-z^{\prime })f_{2}(r)}{r^{3}}\right.\left.+\frac{{\left[{\left(x-x^{\prime }\right)}^{2}+{\left(z+z^{\prime }\right)}^{2}\right]}^{1/2}(z+z^{\prime })f_{2}(r^{\prime })}{{\left(r^{\prime }\right)}^{3}}\right] \end{array},$$

(A7) $$\begin{array}{c} A_{MyHx}=\frac{1}{2\pi \eta }\frac{{\left(x-x^{\prime }\right)}^{2}}{{\left[{\left(x-x^{\prime }\right)}^{2}+{\left(y-y^{\prime }\right)}^{2}\right]}^{1/2}}\left[\frac{\left(z-z^{\prime })f_{1}(r\right)}{r^{4}}\right.\left.+\frac{\left(z+z^{\prime })f_{1}(r^{\prime }\right)}{{\left(r^{\prime }\right)}^{4}}\right]+\frac{jk}{4\pi \eta }\frac{\left(y-y^{\prime }\right)}{{\left[{\left(x-x^{\prime }\right)}^{2}+{\left(y-y^{\prime }\right)}^{2}\right]}^{1/2}}\\ \left[\frac{{\left[{\left(y-y^{\prime }\right)}^{2}+{\left(z-z^{\prime }\right)}^{2}\right]}^{1/2}(z-z^{\prime })f_{2}(r)}{r^{3}}\right.\left.+\frac{{\left[{\left(y-y^{\prime }\right)}^{2}+{\left(z+z^{\prime }\right)}^{2}\right]}^{1/2}(z+z^{\prime })f_{2}(r^{\prime })}{{\left(r^{\prime }\right)}^{3}}\right] \end{array},$$

(A8) $$A_{PzHy}=\frac{jk}{4\pi }\cdot \left(\frac{f_{1}(r)}{r^{2}}+\frac{f_{1}(r^{\prime })}{{\left(r^{\prime }\right)}^{2}}\right)\cdot (x-x^{\prime }),$$

(A9) $$\begin{array}{c} A_{MxHy}=\frac{1}{2\pi \eta }\frac{{\left(y-y^{\prime }\right)}^{2}}{{\left[{\left(x-x^{\prime }\right)}^{2}+{\left(y-y^{\prime }\right)}^{2}\right]}^{1/2}}\left[\frac{\left(z-z^{\prime })f_{1}(r\right)}{r^{4}}\right.\left.+\frac{\left(z+z^{\prime })f_{1}(r^{\prime }\right)}{{\left(r^{\prime }\right)}^{4}}\right]-\frac{jk}{4\pi \eta }\frac{\left(x-x^{\prime }\right)}{{\left[{\left(x-x^{\prime }\right)}^{2}+{\left(y-y^{\prime }\right)}^{2}\right]}^{1/2}}\\ \left[\frac{{\left[{\left(x-x^{\prime }\right)}^{2}+{\left(z-z^{\prime }\right)}^{2}\right]}^{1/2}(z-z^{\prime })f_{2}(r)}{r^{3}}\right.\left.+\frac{{\left[{\left(x-x^{\prime }\right)}^{2}+{\left(z+z^{\prime }\right)}^{2}\right]}^{1/2}(z+z^{\prime })f_{2}(r^{\prime })}{{\left(r^{\prime }\right)}^{3}}\right] \end{array},$$

(A10) $$\begin{array}{c} A_{MyHy}=\frac{1}{2\pi \eta }\frac{\left(x-x^{\prime })(y-y^{\prime }\right)}{{\left[{\left(x-x^{\prime }\right)}^{2}+{\left(y-y^{\prime }\right)}^{2}\right]}^{1/2}}\left[\frac{\left(z-z^{\prime })f_{1}(r\right)}{r^{4}}\right.\left.+\frac{\left(z+z^{\prime })f_{1}(r^{\prime }\right)}{{\left(r^{\prime }\right)}^{4}}\right]-\frac{jk}{4\pi \eta }\frac{\left(x-x^{\prime }\right)}{{\left[{\left(x-x^{\prime }\right)}^{2}+{\left(y-y^{\prime }\right)}^{2}\right]}^{1/2}}\\ \left[\frac{{\left[{\left(y-y^{\prime }\right)}^{2}+{\left(z-z^{\prime }\right)}^{2}\right]}^{1/2}(z-z^{\prime })f_{2}(r)}{r^{3}}\right.\left.+\frac{{\left[{\left(y-y^{\prime }\right)}^{2}+{\left(z+z^{\prime }\right)}^{2}\right]}^{1/2}(z+z^{\prime })f_{2}(r^{\prime })}{{\left(r^{\prime }\right)}^{3}}\right] \end{array},$$

where *k* denotes the propagation constant in free space, and *η* represents the wave impedance in free space. *x*, *y*, and *z* are the coordinates of the measurement point in the Cartesian coordinate system, while *x’*, *y’*, and *z’* denote the coordinates of the dipole in the same coordinate system. *r* is the distance between the measurement point and the equivalent dipole, and *r’* is the distance between the measurement point and the image of the equivalent dipole. For the sake of concise expression, *f*_1_, *f*_2_, *r*, and *r’* are calculated by follows:

(A11) $$f_{1}(d)=\left(1+\frac{1}{jkd}\right)\frac{e^{-jkd}}{d}(d=r,r^{\prime }),$$

(A12) $$f_{2}(d)=\left(1+\frac{1}{jkd}-\frac{1}{k^{2}d^{2}}\right)\frac{e^{-jkd}}{d}(d=r,r^{\prime })$$

(A13) $$r={\left[{\left(x-x^{\prime }\right)}^{2}+{\left(y-y^{\prime }\right)}^{2}+{\left(z-z^{\prime }\right)}^{2}\right]}^{1/2}$$

(A14) $$r^{\prime }={\left[{\left(x-x^{\prime }\right)}^{2}+{\left(y-y^{\prime }\right)}^{2}+{\left(z+z^{\prime }\right)}^{2}\right]}^{1/2}$$
